# Improving quality of life through the routine use of the patient concerns inventory for head and neck cancer patients: main results of a cluster preference randomised controlled trial

**DOI:** 10.1007/s00405-020-06533-3

**Published:** 2020-12-21

**Authors:** Simon N. Rogers, Christine Allmark, Fazilet Bekiroglu, Rhiannon Tudor Edwards, Gillon Fabbroni, Robert Flavel, Victoria Highet, Michael W. S. Ho, Gerald M. Humphris, Terry M. Jones, Owais Khattak, Jeffrey Lancaster, Christopher Loh, Derek Lowe, Cher Lowies, Dominic Macareavy, James Moor, T. K. Ong, A. Prasai, Nicholas Roland, Cherith Semple, Llinos Haf Spencer, Sank Tandon, Steven J. Thomas, Andrew Schache, Richard J. Shaw, Anastasios Kanatas

**Affiliations:** 1 Faculty of Health and Social Care, Edge Hill University, Ormskirk, Liverpool, L39 4QP UK; 2 Liverpool Head and Neck Centre, Liverpool University Hospital Aintree, Liverpool, UK; 3 Leeds Teaching Hospitals and St James Institute of Oncology, Leeds Dental Institute and Leeds General Infirmary, Leeds, UK; 4 Centre for Health Economics and Medicines Evaluation (CHEME), School of Health Sciences, College of Human Sciences, Ardudwy Hall, Normal Site, Bangor University, Bangor, Wales UK; 5 Southway, Guilford, Surrey UK; 6 Liverpool Head and Neck Clinical Trials, Clinical Sciences Building, University Hospital Aintree, Liverpool, UK; 7 School of Medicine, Medical & Biological Sciences, North Haugh, St Andrews, UK; 8 Liverpool Head and Neck Centre, University of Liverpool, Cancer Research Centre, University of Liverpool, 200 London Road, Liverpool, L3 9GA UK; 9 Astraglobe Ltd, Congleton, Cheshire UK; 10 Chair of the Head and Neck Patient and Carer Research Forum, Liverpool Head and Neck Centre, Liverpool University Hospital Aintree, Liverpool, UK; 11 Institute of Nursing and Health Research, Ulster University, Shore Road, Belfast, Newtownabbey, Co, BT37 0QB Antrim, Belfast UK; 12 South Eastern Health and Social Care Upper Newtownards Road, Belfast, BT16 1RH UK; 13 Oral and Maxillofacial Surgery Department, Bristol University, Lower Maudlin Street, Bristol, UK

**Keywords:** Head and neck cancer, Patient concerns inventory, Quality of life, Patient-reported outcomes, Intervention, Randomised trial

## Abstract

**Purpose:**

The patient concerns inventory (PCI) is a prompt list allowing head and neck cancer (HNC) patients to discuss issues that otherwise might be overlooked. This trial evaluated the effectiveness of using the PCI at routine outpatient clinics for one year after treatment on health-related QOL (HRQOL).

**Methods:**

A pragmatic cluster preference randomised control trial with 15 consultants, 8 ‘using’ and 7 ‘not using’ the PCI intervention. Patients treated with curative intent (all sites, disease stages, treatments) were eligible.

**Results:**

Consultants saw a median (inter-quartile range) 16 (13–26) patients, with 140 PCI and 148 control patients. Of the pre-specified outcomes, the 12-month results for the mean University of Washington Quality of Life (UW-QOLv4) social-emotional subscale score suggested a small clinical effect of intervention of 4.6 units (95% CI 0.2, 9.0), *p* = 0.04 after full adjustment for pre-stated case-mix. Results for UW-QOLv4 overall quality of life being less than good at 12 months (primary outcome) also favoured the PCI with a risk ratio of 0.83 (95% CI 0.66, 1.06) and absolute risk 4.8% (− 2.9%, 12.9%) but without achieving statistical significance. Other non-a-priori analyses, including all 12 UWQOL domains and at consultant level also suggested better HRQOL with PCI. Consultation times were unaffected and the number of items selected decreased over time.

**Conclusion:**

This novel trial supports the integration of the PCI approach into routine consultations as a simple low-cost means of benefiting HNC patients. It adds to a growing body of evidence supporting the use of patient prompt lists more generally.

## Introduction

Health-related quality of life (HRQOL) is a key outcome in cancer care. The evaluation of HRQOL is complex with many factors involved [1]. For head and neck cancer (HNC) survivors, HRQOL is not only influenced by site of the tumour, stage and treatment [2] but shaped by patient–clinician relationship, identification of needs, participation in therapeutic alliance, and quality of the rehabilitation service provision [3]. Following treatment, HNC patients have a wide variety and level of unmet needs, with psychological unmet needs being most prevalent [4]. Emotional concerns can be difficult to elicit indicating the importance of undertaking holistic assessments in an attempt to uncover unmet needs [5].

The patient concerns inventory (PCI) was first published in 2009 [6] and is a condition-specific prompt list that allows patients to raise concerns that otherwise might be overlooked [7]. A recent systematic review and content comparison of unmet needs self-report measures used in patients with HNC favoured the PCI compared to 13 other tools [8]. The PCI consists of 56 clinical items and has been used by patients in outpatient clinics, before seeing their consultant. The list guides the outpatient consultation and it covers a range of symptoms and potential problems patients may face after treatment. It has been shown to be feasible in routine consultations [9, 10] and for wider adoption across a cancer network [11]. It is possible to augment the PCI with feedback from the patient as to their HRQOL outcome and one example of this is through the use of the University of Washington questionnaire (UW-QOLv4) [12, 13]. The combination of the PCI and UW-QOL has been shown to be feasible in routine practice and early evidence would suggest that their use in consultations could have a beneficial impact on quality of life [14, 15]. With established cut-offs, it is possible to highlight those doing less well [16]. As outlined in the trial protocol [17] and baseline findings [18], the trial intervention involved a one-page patient summary sheet that was printed following patient completion of the questionnaires on an iPad. This information sheet showed the PCI items flagged, domains of UW-QOL dysfunction, overall QOL, distress thermometer (DT) score and number of health professionals that patients identified as possibly wishing to see, were taken into the consultation with the patient.

The PCI has never before been tested in a randomised trial. Hence, the main aim of this paper is to report the a-priori outcomes of the trial, specifically overall quality of life, social–emotional dysfunction and distress following repeated use of the PCI based summary sheet after a one-year period. Other important outcomes, such as cost-effectiveness, in terms of quality-adjusted life years (QALY), health service use and costs and the cost of the PCI intervention will be reported in a separate paper.

## Methods

The methods have been described in full [18]. The study was a pragmatic cluster-controlled trial conducted at two UK Cancer Centres in Aintree and Leeds, UK. All 15 eligible consultants (the clustering factor) were randomised, 8 to ‘using’ and 7 to ‘not using’ an intervention incorporating the PCI prompt list at all their trial clinics. Consultants with preferences were given their preferred group and those without preference were randomised, so as to limit the possibility of PCI-sceptics dominating the PCI group and PCI-enthusiasts the non-PCI group. Preference-based methodologies have been used in the evaluation of interventions that are about clinical behaviour change to minimise the impact of pre-conceived ideas [19]. Allocation was overseen by the trial medical statistician, before any patient recruitment and was blind to consultant name. In Leeds, three consultants preferred to use the PCI, while three without preference became controls. In Liverpool, three consultants preferred to be controls and the other five without preference were randomised, one as a control and four to using the PCI. A new Aintree consultant, in post soon after the trial began, was randomised to the PCI group. Quality assurance was by initial training and booster sessions for PCI consultants and through a post-consultation survey of PCI patients asking how much the consultant had made reference to the PCI prompt sheet during the consultation.

Eligible patients were treated curatively for primary HNC and included all sites, stages of disease and treatments. Patients treated palliatively or for recurrence, or with history of cognitive impairment, psychoses or dementia were excluded. The first baseline clinic was in April 2017, and to aid recruitment to this novel trial, second primary tumours were accepted from January 2018. Eligible patients were given written information about the trial and willing participants were asked to provide written consent when they next attended hospital. Patients consented to their clinical data being used and to completing research questionnaires before each post-treatment consultation, information from which could be used in their consultation. Neither consultants nor patients were blind to the randomisation, this being a pragmatic trial.

Pre-consultation questionnaires including the PCI prompt list were completed electronically apart from one Liverpool hospital (non-PCI consultant) that used paper. Patient concerns inventory patients took into their consultations a summary sheet of paper that listed (a) all PCI items they selected for discussion, (b) any University of Washington (UWQOL) questionnaire domains in which there was a significant problem or dysfunction, (c) their overall QOL response (d) their DT score and (e) health professionals they wanted to see. This one-page paper summary printout was the visible difference between trial arms as far as contact between consultant and patient was concerned. Control patients completed exactly the same pre-clinic information apart from the PCI prompt list but neither they nor their consultant saw any summary sheet. Both groups completed the EQ-5D-5L for purposes of health economic assessment.

Clinical and demographic data were collected by a baseline questionnaire or by extraction from electronic records. HRQOL data included UW-QOLv4 [12], the DT [20] and EQ-5D-5L [21]. The UW-QOL v4 questionnaire consists of 12 single-item domains, with 3–5 response options scored evenly from 0 (worst) to 100 (best) according to response hierarchy. UW-QOL domains are presented within two subscales, physical function and social–emotional function [22]. The physical function score is the mean of the appearance, swallowing, chewing, speech, taste and saliva domain scores, while the social–emotional score is the mean of the pain, activity, recreation, shoulder, mood and anxiety domain scores. Criteria derived from earlier work can indicate the domains in which patients have a significant problem or dysfunction [16]. A single overall QOL question on the UWQOL v4 asks patients to consider not only physical and mental health, but also other factors, such as family, friends, spirituality or personal leisure activities important to their enjoyment of life; response options are outstanding, very good, good, fair, poor, and very poor.

Trial patients were seen in clinic after treatment as per normal routine. For analysis, ‘intermediate’ (91–273 days) and ‘final’ (≥ 274 days) time windows captured the clinics that fell close to 6 months (183 days) and 12 months (365 days) after the baseline trial clinic. For patients seen more than once in the intermediate window, the closest clinic to 183 days was selected for analysis. In the final window, priority selection was given to patients seen after 12 months and failing this the closest to 365 days. In this paper, we will simply refer to results being ‘at 6 months’ and ‘at 12 months’.

The pre-specified primary outcome measure was the percentage with less than good overall QOL (UWQOLv4) at 12 months. Two pre-specified secondary outcomes were (A) the percentage with a DT score ≥ 4 and (B) the mean social–emotional subscale score of the UWQOLv4. Assuming a control group outcome of 30% for the primary outcome, an intra-cluster correlation (ICC) value of 0.01, a cluster size of 30, and not wishing to miss a halving in outcome, then 312 patients from ≥ 10 consultants were required (with 80% power, 5% level of significance) at 12 months. After factoring in 25% attrition/non-consent, 416 were to be identified at Multidisciplinary Team (MDT) Meetings. For DT ≥ 4, an ICC of 0.01 and a control outcome of 34% anticipated, 294 patients were required at 12 months to avoid missing a halving in outcome. For the social–emotional subscale score, an ICC of 0.025 and a control mean of 75 anticipated, 221 patients were required at 12 months to avoid missing a 10-unit difference in means.

Inference targeted patient outcomes at 12 months. For binary outcomes (overall QOL, DT) binary regression (STATA v13 *binreg* procedure) with the *rr* link option estimated treatment-effect risk ratios/differences, *P* values and 95% confidence intervals, with standard errors robust to intra-cluster correlation obtained using the option ‘cluster’. Logistic regression (STATA *logit*) with robust standard errors and cluster option was also used. Estimates were adjusted for baseline values of the outcome, for consultant clustering and for pre-specified covariates of gender, age (< 55, 55–64, 65–74,  ≥ 75), tumour site (oral cavity, oropharynx, larynx, other), overall clinical grade (early 1–2, advanced 3–4), treatment (surgery only, RT or CT/RT only, surgery with RT or CT/RT,) and free-flap transfer treatment (Yes, No, No surgery). Random effects linear regression (STATA *xtreg* procedure) estimated the treatment-effect difference in UWQOL socio-emotional mean subscale scores, with adjustments for baseline subscale scores (quintile categories), for consultant clustering and for the pre-specified covariates. Standard errors were estimated by a cluster bootstrap that resampled with replacement over consultant clusters. In non-apriori analyses, the UWQOL physical function subscale score and all 12 UWQOL domain scores were analysed in the same way using random effects linear regression. Quintile categories were created for baseline physical function subscale scores and a three-level categorisation into best score (100), dysfunction, and in-between these extremes was used for baseline UWQOL domain scores. We used the estimator provided by the *loneway* command in STATA v13 to estimate outcome ICCs.

The PCI trial had ethical approval from North West-Liverpool Central Research Ethics Committee REC reference: IRAS 16/NW/0465, Project ID: 189,554. It also had approval from the Health Research Authority (HRA).

The study closed formally on the 30 June 2020 following lockdown due to COVID-19 in England on the 24 March 2020.

## Results

**Fig. 1:**
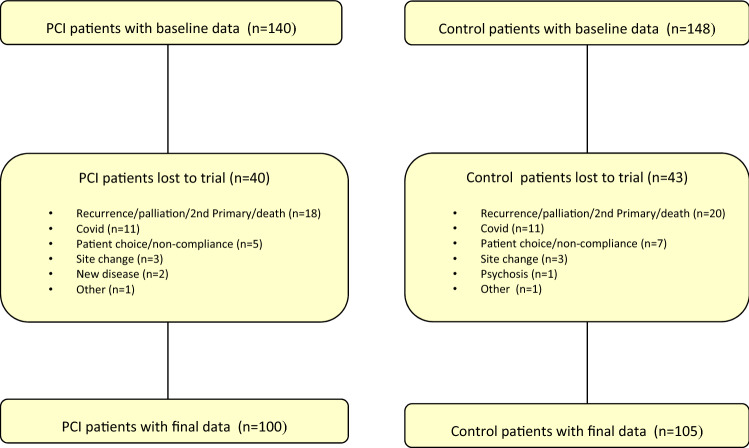
Patient flow from trial baseline to final clinic

Fifteen consultants were eligible and all participated throughout the trial seeing a median [Inter-Quartile Range (IQR)] of 16 (13–26) patients, range 5–48. Baseline clinics ran from April 2017 to October 2019 with 140 intervention PCI and 148 control patients and a total of 1186 trial clinic appointments. Median IQR number of clinics for PCI patients was 4 (3–5), range 1–10, and for controls 4 (3–6), range 1–10. A detailed patient flow chart from MDT to baseline clinic has been published [18]. Figure 1 shows the flow chart from baseline clinic through to completion of the trial. There were final clinic data for 71% of patients in each trial group, median IQR 69% (56–82%) for the 15 consultants. Nearly half (46%) of the patient attrition was due to cancer recurrence, palliation, 2nd primary and death, with 5 patients dying (2 PCI, 3 control). One-quarter (27%) of the overall loss was due to early closure of the trial because of the COVID-19 pandemic; only 14% was due to patient choice/non-compliance. Final trial clinics (referred to as at 12 months) were a median IQR of 357 (329–380) days after baseline for 100 PCI patients and 364 (322–396) days for 105 controls; intermediate clinics (at 6 months) were 182 (147–210) days, *n* = 113 and 175 (147–196) days, *n* = 126, respectively.

Baseline characteristics of PCI and control groups have already been described [18]; briefly, the two trial groups were similar in demographic and clinical characteristics as well as in HRQOL measures apart from differences in tumour location, tumour staging and mode of treatment. These exceptions were cluster (consultant) related with MFU and ENT consultants seeing different types of cases. Baseline characteristics of patients with final outcome data can be seen in Table 1, and apart from tumour site and mode of treatment, the balance between PCI and control groups was broadly similar. Overall loss to follow-up was higher in patients living alone, not working, in households receiving benefits and living in more deprived neighbourhoods. It was also higher in those with worse HRQOL and in those having free-flap transfer surgery followed by adjuvant therapy.

**Table 1:** Baseline characteristics of those with final data, and loss to follow-up

	Patients with final data		Patients without final data		Overall loss to follow-up	*p* value^*^
	PCI	Control	PCI	Control	%	
All patients	100	105	40	43	29	
Site						
Aintree	55	64	27	32	33	0.05
Leeds	45	41	13	11	22	
Gender						
Female	35	27	14	14	31	0.58
Male	65	78	26	29	28	
Age at baseline clinic						
< 55	20	29	9	13	31	0.68
55–64	45	42	17	12	25	
65–74	23	23	9	12	31	
≥ 75	12	11	5	6	32	
Tumour site						
Oral cavity	38	53	17	26	32	0.08
Oropharynx	33	37	9	12	23	
Larynx	17	8	13	3	39	
Other	12	7	1	2	14	
Overall stage						
Early 0–2	40	49	16	19	28	0.90
Advanced 3–4	60	56	24	24	29	
Primary treatment^**^						
S only	36	37	10	12	23	0.06
S only & FF	5	12	2	2	19	
RT or RT/CT only	26	13	12	7	33	
S & (RT or RT/CT)	24	27	7	10	25	
S & (RT or RT/CT) & FF	9	16	9	12	46	
WHO comorbidity						
0	65	63	23	28	28	0.28
1	23	28	5	11	24	
2–4	12	14	12	4	38	
ACE27 comorbidity						
None	58	49	13	17	22	0.08
Mild	26	35	15	19	36	
Moderate/severe	16	21	12	7	33	
Living situation in house/flat						
Alone	18	21	11	15	40	0.03
With others	82	83	29	26	25	
Working						
Yes	41	31	7	9	18	0.01
No	55	73	31	33	33	
Financial benefits (household)						
Yes	30	38	19	20	36	0.007
No	64	61	14	19	21	
Smoking habit						
Current	12	12	4	9	35	0.60
Former	60	57	21	25	28	
Never	26	33	12	9	26	
Alcohol habit						
Current	80	66	20	28	25	0.08
Former	15	30	15	13	38	
Never	4	6	1	2	23	
IMD 2019 quintile						
1 = least deprived	12	14	4	4	24	0.01
2	23	22	6	4	18	
3	18	22	4	5	18	
4	12	10	5	13	45	
5 = most deprived	35	37	21	17	35	
UWQOL Overall Quality of life						
Good, V good, Outstanding	70	77	25	27	26	0.16
Fair, Poor, V poor	30	28	15	16	35	
Distress thermometer (DT) score						
0–3	55	67	19	18	23	0.03
4–10	45	38	21	25	36	
UWQOL social-emotional subscale score (quintiles)						
< 55.8	10	19	10	18	49	0.002
55.9–70.0	21	22	9	10	31	
70.1–81.7	25	20	7	3	18	
81.8–90.8	18	16	8	5	28	
90.9–100	26	28	6	7	19	

*S* Surgery, *RT* Radiotherapy,*CT* Chemotherapy, *FF *Free flap transfer

*Fishers exact test

**Fig. 2:**
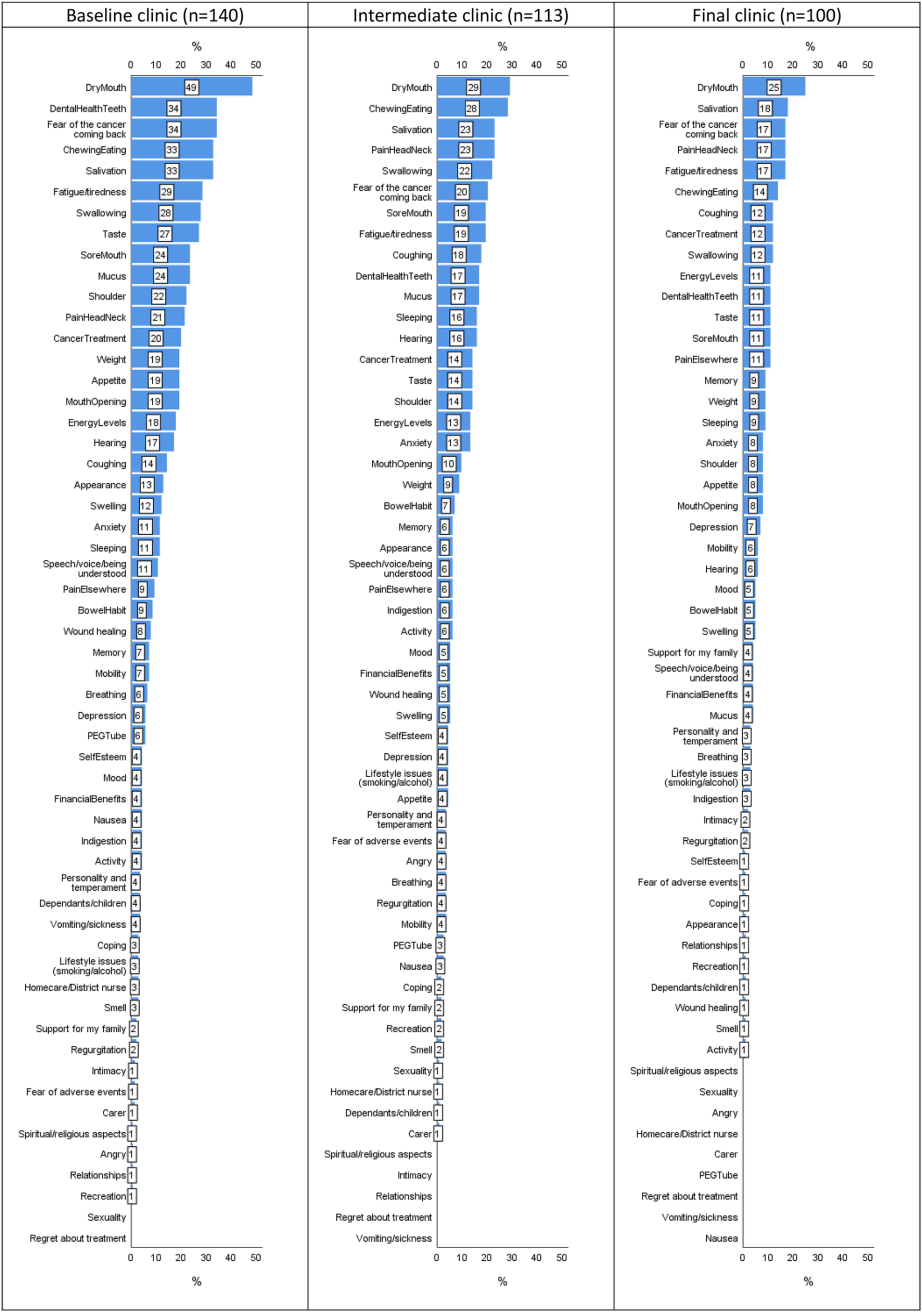
PCI items selected at baseline, intermediate and final clinics

The median IQR number of PCI items was 5 (2–9) at baseline, reducing to 3 (1–7) at 6 months and 2 (0–4) at 12 months. Dry mouth was the most frequent item selected, 49% at baseline, 29% at 6 months and 25% at 12 months (Fig. 2). Other items most selected throughout the trial were fear of the cancer coming back, chewing/eating, salivation, fatigue/tiredness and pain in the head/neck. The percentage of patients selecting one or more health professionals they wanted to see was 46% (65/140) at baseline, 31% (35/113) at 6 months and 18% (18/100) at 12 months. Professionals most selected at baseline were dentist (16%, 22), surgeon (14%, 19), radiotherapist/oncologist (9%, 12), Speech & Language therapist (8%, 11) and dental hygienist (8%, 11). In the post-consultation questionnaire, the vast majority of PCI patients said that their consultant had made ‘a great deal’ of reference to the PCI prompt sheet during the consultation, 88% (117/133) at baseline, 90% (97/108) at 6 months and 93% (91/98) at 12 months. In most other instances (13/133, 7/108, 5/98), consultants had ‘somewhat’ referenced the prompt sheet, and rarely had they made ‘a little’ (3, 3, 1) reference or ‘not at all’ (0, 1, 1). Median IQR consultation times at baseline were 11 (8–15) minutes for 138 PCI and 10 (7–13) minutes for 148 control patients; at 6 months: 10 (7–12) minutes for 112 PCI and 10 (7–11) minutes for 126 controls; at 12 months: 8 (7–11) minutes for 97 PCI and 9 (6–12) minutes for 103 controls.

Results for the primary outcome measure, i.e. the percentage of patients at 12 months with overall QOL that was less than good, favoured the PCI intervention though these results were not statistically significant (Table 2). In the PCI group, the percentage fell from 30% at baseline to 22% at 12 months whilst in the control group, it fell from 27 to 25%. After adjustment for baseline outcome, consultant clustering, tumour site and treatment (including free-flap transfer), the estimate of risk ratio at 12 months was 0.83 (95% CI 0.66, 1.06), *p* = 0.14. Similar adjustments regarding absolute risk difference gave an estimate of 4.8% in favour of PCI over control with 95% confidence interval ranging from 2.9% against PCI to 12.9% for PCI. Results for the percentage of patients at 12 months with a DT score ≥ 4 did not favour either group after adjustment (Table 3); in the PCI group, the percentage fell from 45% at baseline to 33% at 12 months whilst in the control group, it fell from 36% to 30%. However, it was not possible to further adjust either binary measure for age, gender or overall clinical stage because of convergence issues. Estimation of odds ratios gave a similar progression in regard to the primary outcome (0.86, 0.77, 0.75 with 0.78 after further adjustment for age, gender and stage) and for the DT (1.12, 1.00, 1.18 and then 0.99). Results for the other a-priori outcome, the UWQOL social–emotional subscale mean score at 12 months, also favoured the PCI intervention and these results were statistically significant (Table 4), with the best estimate suggesting a small clinical effect of 4.6 units (95% CI 0.2, 9.0), *p* = 0.04 after full adjustment.

**Table 2:** Pre-specified primary outcome: less than good overall QOL at 12 months

	Baseline		6 months		12 months		Risk ratio at 12 months, with 95% confidence interval and *p* value		
							Unadjusted	Adjusted for baseline QOL & consultant clustering^a^	Adjusted also for tumour site, treatment & free-flap
	%	*n*	%	*n*	%	*n*			
Data at baseline and in 12 month window									
PCI	30	30/100			22	22/100	0.89 (0.54, 1.46) *p* = 0.64	0.85 (0.59, 1.24) *p* = 0.40	0.83 (0.66, 1.06) *p* = 0.14
Control	27	28/105			25	26/105			
All available data									
PCI	32	45/140	24	27/113	22	22/100			
Control	30	44/148	24	30/126	25	26/105			
Data at baseline and in both 6 and 12 month windows									
PCI	29	26/90	21	19/90	22	20/90			
Control	26	26/101	19	19/101	24	24/101			

^a^*ICC* Intra-class correlation estimated as Zero, with 95% confidence interval (0, 0.058)

**Table 3:** Pre-specified secondary outcome: distress thermometer (DT) score ≥ 4 at 12 months

	Baseline		6 months		12 months		Risk ratio at 12 months, with 95% confidence interval and *p* value		
							Unadjusted	Adjusted for baseline DT & consultant clustering^a^	Adjusted also for tumour site, treatment & free-flap
	%	*n*	%	*n*	%	*n*			
Data at baseline and in 12 month window:									
PCI	45	45/100			33	33/100	1.08 (0.73, 1.62) *p* = 0.70	0.93 (0.60, 1.46) *p* = 0.76	1.04 (0.68, 1.59) *p* = 0.85
Control	36	38/105			30	32/105			
All available data									
PCI	47	66/140	41	46/113	33	33/100			
Control	43	63/148	37	47/126	30	32/105			
Data at Baseline and in both 6 and 12 month windows:									
PCI	46	41/90	38	34/90	34	31/90			
Control	36	36/101	37	37/101	29	29/101			

^a^*ICC* Intra-class correlation estimated as 0.00345, with 95% confidence interval (0, 0.064)

**Table 4:** Pre-specified secondary outcome: UWQOL social-emotional subscale score at 12 months

	Baseline		6 months		12 months			Difference between means at 12 months, with 95% confidence interval and *p* value		
								Unadjusted	Adjusted for baseline value^a^ & consultant clustering^b^	Adjusted also for tumour site, treatment, free-flap, clinical stage, age & gender
	Mean (SD)	*n*	Mean (SD)	*n*	Mean (SD)		*N*			
Data at baseline and in 12 month window										
PCI	77.1 (16.0)	100				83.8 (15.9)	100	6.0 (1.3, 10.7) *p* = 0.01	3.7 (0.3, 7.1) *p* = 0.03	4.6 (0.2, 9.0) *p* = 0.04
Control	73.4 (20.3)	105				77.8 (18.7)	105			
All available data										
PCI	75.0 (17.8)	140	79.4 (17.3)	113		83.8 (15.9)	100			
Control	70.5 (21.0)	148	75.7 (19.4)	126		77.8 (18.7)	105			
Data at baseline and in both 6 and 12 month windows										
PCI	76.3 (16.1)	90	81.0 (16.5)	90		83.3 (16.0)	90			
Control	74.3 (19.2)	101	78.2 (18.1)	101		78.4 (18.4)	101			

*SD* Standard deviation. The subscale score ranges from 0 to 100 with lower scores indicating worse QOL

^a^Baseline value: Quintile categorisation (see Table 1)

^b^*ICC *Intra-class correlation estimated as 0.03191, with 95% confidence interval of (0, 0.111)

Since the subscale score is the average of six domain scores, a set of extra analyses were done for each domain (Table 5), and all of these analyses favoured the PCI intervention, with a mix of small effects suggested for each domain. Similar analyses of the UWQOL physical function subscale mean score at 12 months, and its six component domains, also favoured the PCI intervention with small effects observed throughout. The full response range for the overall QOL (Table 6) indicated a tendency within each level of QOL at baseline for the PCI group to have a better set of QOL responses at 12 months; overall QOL improved for 42% (42) of PCI patients and 30% (31) of control patients. Table 7 provides a more simplistic cluster-level descriptive summary of the trial data. Six of the 8 PCI consultants saw a reduction in the number of their patients having less than good overall QOL, in contrast to 3 of the 7 control consultants. The changes in mean socio-emotional subscale scores tended to be larger in patients under PCI consultants (median 7.5) than control consultants (median 3).

**Table 5:** Other UWQOL outcomes (not pre-specified) for 100 PCI and 105 control patients with data at baseline and at 12 months

	Mean (SD)				Difference between group means at 12 months, with 95% confidence interval and *p* value		
	Baseline		12 months		Unadjusted	Adjusted for baseline value^a^ and consultant clustering	Adjusted also for tumour site, treatment, free-flap, clinical stage, age and gender
	PCI	Control	PCI	Control			
UWQOL social–emotional subscale domain scores							
Pain	73 (26)	71 (29)	88 (20)	80 (27)	7.5 (1.1, 13.9)	7.3 (2.0, 12.6)	7.5 (1.9, 13.2)
Activity	74 (23)	70 (24)	82 (21)	76 (24)	6.3 (0.1, 12.5)	5.0 (0.8, 9.3)	6.8 (0.8, 12.8)
Recreation	83 (22)	75 (23)	86 (19)	79 (22)	6.5 (0.9, 12.0)	3.8 (− 0.9, 8.5)	3.1 (− 2.4, 8.6)
Shoulder	82 (26)	73 (33)	87 (25)	81 (28)	5.3 (− 2.1, 12.6)	2.8 (− 2.4, 8.0)	4.7 (− 0.8, 10.1)
Mood	77 (23)	74 (27)	83 (22)	77 (24)	6.1 (− 0.2, 12.4)	5.2 (1.7, 8.6)	4.2 (− 0.7, 9.1)
Anxiety	74 (26)	77 (26)	78 (23)	74 (28)	4.5 (− 2.4, 11.4)	6.1 (0.0, 12.1)	6.9 (− 0.2, 14.0)
UWQOL Physical function subscale score	71 (17)	69 (20)	81 (15)	76 (20)	5.7 (0.7, 10.6)	4.4 (1.5, 7.3)	4.5 (0.6, 8.4)
UWQOL physical function subscale domain scores							
Appearance	80 (17)	73 (20)	89 (14)	83 (19)	5.2 (0.6, 9.7)	3.2 (− 0.3, 6.6)	2.2 (− 1.4, 5.8)
Swallowing	78 (22)	76 (23)	86 (16)	80 (25)	5.7 (0.0, 11.4)	4.6 (− 0.1, 9.2)	5.6 (− 0.5, 11.7)
Chewing	65 (31)	67 (32)	80 (27)	75 (32)	4.7 (− 3.3, 12.8)	5.7 (− 1.3, 12.7)	6.2 (− 3.5, 15.9)
Speech	83 (18)	79 (21)	90 (15)	83 (20)	6.9 (2.1, 11.7)	4.9 (0.7, 9.2)	4.3 (0.7, 8.0)
Taste	64 (32)	61 (35)	77 (28)	70 (32)	6.9 (− 1.3, 15.1)	6.8 (0.6, 12.9)	6.7 (− 0.4, 13.7)
Saliva	58 (32)	57 (33)	67 (32)	62 (31)	4.5 (− 4.1, 13.1)	3.9 (− 2.0, 9.9)	2.3 (− 5.2, 9.8)

*ICC* Intra-class correlation for physical subscale score estimated as 0.0125, with 95% confidence interval of (0, 0.0786)

^a^Baseline value: Quintile categorisation for UWQOL Physical function; 3-level categorisation into best score, dysfunction, and in-between these extremes for the 12 UWQOL domains

**Table 6:** Change in overall QOL from baseline to 12 months

Category Change^a^	Baseline	Final	PCI (*N* = 100)	Control (*N* = 105)
+ 4	V poor	V good	1	–
+ 1	V poor	Poor	2	–
0	V poor	V poor	1	2
+ 3	Poor	V good	2	–
+ 2	Poor	Good	–	1
+ 1	Poor	Fair	2	2
0	Poor	Poor	1	1
+ 3	Fair	Outstanding	1	–
+ 2	Fair	V good	3	2
+ 1	Fair	Good	9	10
0	Fair	Fair	8	8
− 1	Fair	Poor	–	1
− 2	Fair	V poor	–	1
+ 1	Good	V good	20	14
0	Good	Good	10	13
− 1	Good	Fair	4	5
− 2	Good	Poor	–	1
+ 1	V good	Outstanding	2	2
0	V good	V good	19	23
− 1	V good	Good	4	9
− 2	V good	Fair	3	2
− 3	V good	Poor	–	2
0	Outstanding	Outstanding	1	2
− 1	Outstanding	V good	4	2
− 2	Outstanding	Good	2	1
− 3	Outstanding	Fair	–	1
− 4	Outstanding	Poor	1	–

^a^The UWQOL Overall QOL question has 6 category responses: Outstanding, Very good, Good, Fair, Poor, Very poor

**Table 7:** Summary results by consultant of the 205 patients with baseline & 12 month data

	Consultant	Patients		Baseline casemix (*n* = 205)										
		Baseline	12 months	Female	Mean age	Stage 3–4	Overall cavity	Oropharynx	Larynx	Other	Surgery only	RT or RT/CT	Surgery & RT or RT/CT	Free-flap
PCI	1	23	17	10	62	6	17	–	–	–	10	–	7	7
	2	16	11	4	60	9	–	7	4	–	2	3	6	–
	3	16	9	2	64	6	–	5	4	–	3	2	4	–
	4	28	24	5	63	16	1	11	7	5	8	10	6	–
	5	15	13	6	65	6	10	1	1	1	7	1	5	5
	6	15	8	1	58	8	–	6	–	2	3	4	1	–
	7	9	9	3	59	8	2	3	1	3	–	6	3	–
	8	18	9	4	63	1	8	–	–	1	8	–	1	2
Control	1	17	11	3	60	7	9	2	–	–	4	1	6	4
	2	13	12	5	62	3	9	1	–	2	8	–	4	6
	3	6	4	2	56	2	4	–	–	–	3	–	1	2
	4	48	37	5	60	29	1	27	6	3	7	9	21	1
	5	33	25	10	60	9	20	3	–	2	16	–	9	12
	6	26	11	2	60	5	10	3	–	–	9	2	2	3
	7	5	3	0	76	1	–	1	2	–	2	1	–	–
	PCI	140	100	35	63	60	38	33	17	12	41	26	33	14
	Control	148	105	27	60	56	53	37	8	7	49	13	43	28

	Consultant	Overall QOL Less than good			Distress thermometer ≥ 4			Mean socio-emotional subscale score		
		Baseline	12 Months	Change	Baseline	12 Months	Change	Baseline	12 Months	Change^a^
PCI	1	5	4	− 1	5	10	+ 5	78	82	5
	2	2	–	− 2	7	4	− 3	82	92	10
	3	3	2	− 1	6	2	− 4	70	81	11
	4	9	7	− 2	9	7	− 2	77	81	3
	5	5	3	− 2	4	5	+ 1	80	83	3
	6	3	2	− 1	4	1	− 3	78	86	9
	7	2	3	+ 1	5	2	− 3	68	83	16
	8	1	1	0	5	2	− 3	82	88	6
Control	1	4	5	+ 1	3	5	+ 2	68	74	6
	2	4	2	− 2	4	4	0	67	76	8
	3	–	1	+ 1	2	–	− 2	92	94	2
	4	9	6	− 3	10	9	− 1	78	80	1
	5	11	9	− 2	12	10	− 2	62	72	10
	6	–	3	+ 3	6	4	− 2	82	81	-1
	7	–	–	0	1	–	− 1	87	90	3
	PCI	30	22	− 8	45	33	− 12	77	84	7
	Control	28	26	− 2	38	32	− 6	73	78	4

^a^Changes based on mean scores at 2 decimal places before rounding

## Discussion

Interventions aimed at improving the quality of life (QoL) of HNC survivors are of critical importance when considering the increasing number of survivors and the significant life-long treatment burden for some patients who have poor functional, emotional and social outcomes. It is essential to provide robust evidence from randomised trials about the effectiveness of psychologically-based interventions intended to improve QoL and its subscales. Thus far, such intervention studies in HNC patients have produced insufficient data to support their effectiveness for improving quality of life [23–27]. However, there is evidence of benefit in other cancers, for example, a randomised trail reporting symptom monitoring with patient-reported outcomes during routine cancer treatment [28]. In addition, van der Meulen [29] reported that nurse-led psychosocial intervention between 12 and 24 months after HNC treatment did make a significant improvement in emotional and physical functioning, pain, swallowing, social contact, mouth opening and depressive symptoms.

The outpatient consultation provides an ideal opportunity for integration of a prompt list intervention. The PCI can be routinely integrated into clinical care [9, 10], and a holistic patient-centred approach seems appropriate anyway regardless of any significant findings. Furthermore, it is something much appreciated and widely accepted by most patients [11]. Its use adds an element of quality assurance particularly when the patient is less familiar to the consultant or is being reviewed by a more junior member of staff. The consultants’ training focused on ensuring that issues identified by the patient were considered and if there were too many issues raised, there was then an agreement with the patient to focus on three or four they felt most important. The post-consultation questionnaire confirmed that the patients felt the PCI had been used throughout the trial.

The purpose of this paper was to focus on a number of outcomes that were specified in advance and for which sample size calculations were made. Variables used to adjust the analyses were also specified in advance, and included tumour site, stage and treatment. Therefore, the main confidence interval estimations can be interpreted without concern about post hoc selection. Other analyses should be regarded as exploratory. Our findings should have generalisability given that all eligible consultants from the two hospitals participated throughout the trial. In addition, there was good recruitment and sustainability throughout the trial in those patients often hard to reach, such as the elderly and those in more socially deprived groups. The choice of a cluster design is a strength of the study, the rationale behind which was discussed in detail in the baseline results paper [18]; individual patient randomisation was ruled out because of the likely sensitization of consultants to using the PCI, which could have led to certain strategies being carried over to when control group patients were being seen. A further strength of the trial was the relatively small proportion of refusals and withdrawals through patient choice, and the number missed for logistical reasons by trial staff was also small. Another strength was the electronic data capture which, apart from the paper-based baseline questionnaire, virtually eliminated missing data in the outpatient clinic setting.

From this study, in terms of the primary outcome of less than good overall QOL at 12 months, the best estimate after various adjustments, including baseline level, was an absolute benefit at 12 months of about 5% from the PCI intervention and a risk ratio of 0.83 which suggests about one in six patients might benefit. Interpretation, however, is made difficult due to wide confidence intervals that include no benefit at all.

Perhaps where the PCI approach is helping most for patients is through the social–emotional aspect of cancer recovery [30]. Although the benefit from PCI intervention of 4.6 units in the social–emotional subscale score at 12 months after adjustment seems small, it is clinically meaningful [22]. It is of note also that results for all 12 UW-QOL domains favoured the PCI with a small benefit (Table 5), with improvements also seen for both groups over time which probably reflects adaption over the year. Fear of recurrence is frequently raised by patients on the PCI (Fig. 2) and is recognised as a major concern over many follow-up consultations [31]. It remains to be fully evaluated but the hypothesis is that the prompt allows the patient permission to talk about this aspect and seek reassurance or further information. The PCI approach might be very helpful for those HNC patients likely to take a more passive role in medical consultations, such as patients of lower socio-economic strata [32].

Previous studies [22] have shown that the domain and subscale scores of the UW-QOL, notably the social–emotional subscale, correlate with overall QoL. It is possible that the simplicity of the binary primary overall QOL outcome in this trial diluted the benefit patients gained from this intervention. When analysing the change in overall QOL across all six-response options for overall QOL, the PCI group appeared to do slightly better (Table 6). Given the variation in numbers of patients by consultant and variation in types of patients seen and treated, it was reassuring to see that the analyses at consultant level (Table 7) provided some support, be it at a rather simplistic summary level, of the main findings reported at patient level. Whereas the unadjusted results for the DT (Table 3) tended to favour the PCI, as did the results after adjustment for baseline value and clustering, the results after full adjustment did not favour either group. No trial of this size stands alone as evidence and further trials would provide reassurance of the positive indications suggested by this trial.

The inclusion of the PCI does not significantly lengthen the consultation time since in comparison to the control group the first trial PCI consultations only took one minute longer on average whilst at 12 months they were one minute shorter.

One limitation of the trial was that it was under-powered, in part from a greater loss of patients than expected after identifying likely patients at the MDT and before the baseline trial clinic; half of this loss comprised patients who could not possibly have started the trial, for example, because of death, recurrence, palliation, changes in travel to non-trial sites and for other reasons, such as being in another trial and having mental health issues [18]. Further losses between baseline and the final trial clinic (Fig. 1) were predominantly for clinical reasons or because of COVID-19. Pragmatically, we accept that “size and power are irrelevant once the experiment has actually been carried out. At this point, the trial is analysed using confidence intervals to show the plausible values for the treatment effects” [33]. One unavoidable limitation was the lack of blinding but this is a distinctive feature of pragmatic cluster trials. Since recruitment of patients took place after the randomisation of clusters, there was always a possibility of selection bias, but this was minimised because patients were allocated to individual consultants through the cancer tracking referral process without knowing which consultants used PCI and which did not.

The focus of the project is now related to wider implementation of the PCI approach in clinical care, research around the mechanisms of action, ways of improving efficacy, and education resources for patients and clinicians. An implementation phase could include aspects of patient–clinician communication and patient empowerment. It would be interesting to apply the intervention earlier in the follow-up of patients as perhaps there might be more benefit, possibly starting in the pre-treatment phase. To achieve this, appropriate IT infrastructure is required to allow patients to access the PCI tool prior to their consultation and allow this to be integrated into clinical care. This needs to be part of an integrated strategy of engaging patients using IT technology. The need for IT solutions around COVID-19 might hasten the breakdown of barriers previously encountered by both patients and clinicians in respect of IT.

## Conclusion

In summary, study is the first randomised controlled trial to evaluate the benefit of the PCI approach. Notable strengths of this study were the originality (landmark trial) with 15 consultants in a routine NHS outpatient setting, its clinical significance as a low-cost intervention and a strong contribution made by patients themselves to the design and delivery of the trial. Clinicians find the PCI straightforward to use, with minimal training, with the vast majority of patients appreciating the approach and wishing to continue to use it in the future. The study suggests a small but meaningful benefit in outcomes from this PCI approach using routine care. Such interventions are increasingly important given the rising incidence of HNC, more people living longer with the burden of treatment-related issues and the importance of addressing unmet needs in the early post-treatment survivorship phase.

## Abbreviations

DT: Distress thermometer
EQ-5D-5 L: EuroQol 5 dimension questionnaire
HNC: Head and neck cancer
HRQOL: Health-related quality of life
MDT: Multi-professional team
PCI: Patients concerns inventory
PCI-HN: Patient concerns inventory-head neck
QOL: Quality of life
RCT: Randomised controlled trial
UWQOL: University of Washington QOL questionnaire
UWQOLv4: University of Washington QOL questionnaire version 4
